# Mitochondrial microprotein MOCCI controls neuroinflammation by altering glial activation states

**DOI:** 10.3389/fimmu.2026.1798798

**Published:** 2026-07-09

**Authors:** Kimberle Shen, Ke Guo, Sze Huey Leong, Radiance Lim, Wei Leong Chew, Cheryl Q. E. Lee, Lena Ho

**Affiliations:** 1 Genome Institute of Singapore (GIS), Agency for Science, Technology and Research (ASTAR), Singapore, Singapore; 2 Cardiovascular Metabolic Disorders Program, Duke-National University of Singapore (NUS) Graduate School, Singapore, Singapore; 3 Synthetic Biology Translational Research Programme, Yong Loo Lin School of Medicine, National University of Singapore, Singapore, Singapore

**Keywords:** astrocytes, C15orf48, glia, glia activation, microglia, MOCCI, neuroinflammation

## Abstract

Metabolic regulation and its underlying mechanisms play a critical role in controlling and resolving inflammation in the brain, directly shaping glial cell activation and the central nervous system’s response to injury and disease. In our screen for microproteins that modify inflammatory outcomes, we discovered MOCCI (protein product of C15orf48/AA467197) as a significant regulator of gut and lung inflammation. However, its involvement in neuroinflammation is unknown. Here, we show that MOCCI is upregulated in microglia and astrocytes in both the mouse and human brain upon inflammation, and is required for orchestrating proper, complete, and beneficial activation of microglia and astrocytes. Induction of MOCCI triggers the transition of glia into a neuroprotective state and promotes the resolution of inflammation. *In vitro*, MOCCI deficiency leads to reduced migration, phagocytosis and cytokine secretion in microglia and astrocytes. In the cuprizone mouse model of multiple sclerosis, MOCCI plays a role in both demyelination and remyelination. These results position MOCCI as a molecular brake on neuroinflammation, highlighting its therapeutic potential for targeting glial metabolic health and resolving chronic CNS inflammation in neurodegenerative disease.

## Introduction

Neurodegenerative diseases are becoming increasingly prevalent as the population ages and are a significant burden on individuals and society, yet few therapies exist due to the complexity and poorly understood nature of these diseases.

Recent studies highlight neuroinflammation as a central driver, rather than a bystander effect, in the progression of neurodegenerative disorders such as Alzheimer’s disease (AD), Parkinson’s disease (PD), multiple sclerosis (MS), and amyotrophic lateral sclerosis (ALS) (1–3). Glial cells, such as astrocytes and microglia, are key players in the maintenance of central nervous system (CNS) homeostasis. In response to insults such as protein aggregates, oxidative stress or cellular injury, microglia and astrocytes become activated. Activated glia promote phagocytic clearance of pathogens, abnormal proteins and cellular debris which leads to the resolution of inflammation and the start of regeneration (4). However, they can also trigger neurotoxic cascades, neuronal death and the formation of astrocytic glial scars through over-exuberant release of pro-inflammatory cytokines. Glial activation is thus a double-edged sword, which, in some contexts, can contribute towards a vicious cycle of neuroinflammation and neurodegeneration. Understanding the delicate relationship between glial activity and neuroinflammation is thus critical for developing targeted therapeutic interventions aimed at modulating neuroinflammatory responses to halt the progression of neurodegenerative diseases.

Traditionally, activated microglia are classified into two opposing groups: M1 (classical activation; pro-inflammatory), and M2 (alternative activation; anti-inflammatory). An imbalance between these two phenotypes is thought to underlie neurological and neurodegenerative conditions (5, 6). Likewise, reactive astrocytes are classed into A1 (pro-inflammatory) and A2 (anti-inflammatory) astrocytes, analogous to their M1 and M2 counterpart (7, 8). A1 astrocytes are a reactive phenotype known for promoting neuroinflammation and neurotoxicity, often seen in various neurodegenerative conditions. In contrast, A2 astrocytes exhibit neuroprotective functions by releasing neurotrophic and anti-inflammatory factors, supporting neuronal survival and repair mechanisms following CNS injuries. Since the advent of single cell and imaging technologies, recent studies have highlighted the transcriptomic, morphological and functional heterogeneity of astrocyte and microglia populations. Instead of an A1 and A2, M1 and M2 dichotomy, and the belief that all astrocytes and microglia populations reside on a spectrum between two distinct states, recent literature has reflected a shift towards a paradigm where there are multiple reactive profiles and activation states (9, 10). It is possible that different profiles are induced by different types of injury (e.g. ischemic injury (11) vs viral infection (12)), or that they reside in different parts of the brain.

Mitochondrial dysfunction is also implicated in most neurodegenerative disorders (13). Mechanisms connecting the two include impairment of autophagy, ROS production favoring the accumulation of abnormal protein aggregates, synaptic toxicity due to mitochondrial deficiency network system, and defective protein quality control by the proteasome system (14). The CNS is also particularly vulnerable to mitochondrial dysfunction due to its high energetic requirements. In PD, GWAS-identified risk loci include PARK genes, which are required to maintain mitochondrial quality through mitophagy (15). In MS, an autoimmune neurodegenerative disorder, glial cells actively participate in the onset and progression of the disease, and exhibit mitochondrial defects, such as abnormal morphology, energetic dysfunction and defects in redox reactions (16). As such, recent studies suggest the use of mitochondrial transfer as a therapeutic approach for CNS disorders. Mitochondrial dysfunction is also linked to neuroinflammation: mitochondrial-derived damage-associated molecular patterns (DAMPs) such as mtDNA and mtRNA can trigger neuroinflammation, while inflammatory factors released by microglia and astrocytes can affect mitochondria function. Mitochondria in activated immune cells also undergo metabolic reprogramming, to meet the energetic demands associated with immune cell activation and effector functions such as migration and phagocytosis (17–19).

Mitochondrial microproteins, which are small proteins produced in the mitochondria, have been shown to play a role in regulating inflammation, including neuroinflammation (20). Mitochondrial peptides encoded by mitochondrial DNA (mtDNA), such as Humanin, are synthesized directly within the mitochondria and are often integral to mitochondrial function, playing crucial roles in managing cellular stress responses and maintaining mitochondrial integrity (21). In contrast, nuclear-encoded mitochondrial peptides, like Miro-1, are synthesized in the cytoplasm and then imported into the mitochondria. These peptides influence mitochondrial dynamics, including fission, fusion, and transport, and play a role in cellular signaling pathways, contributing to cellular homeostasis and neuroprotection. Our previous work identified Modulator of cytochrome C oxidase during Inflammation (MOCCI), in a screen for mitochondrial microproteins that modify inflammatory outcomes (22). MOCCI is upregulated during inflammation, reduces cytokine and reactive oxygen species (ROS) production, and promotes resolution by dampening the immune response. MOCCI is the protein product encoded by the C15orf48/AA467197 gene. For consistency and clarity, in this manuscript we refer to the MOCCI/C15orf48/AA467197 knockout as C15orf48-KO, AA467197 as the mouse transcript, and MOCCI to denote the gene product, encompassing both RNA and protein. We adopt this convention because the knockout disrupts the gene as a whole and cannot distinguish whether the observed effects are from the loss of the RNA, the protein, or both.

Here, we examine the role of MOCCI in modulating neuroinflammation. We find that MOCCI mediates a switch in the activation profile of astrocytes and microglia in two neuroinflammation disease mouse models and controls key cellular processes in immune response such as phagocytosis, migration and cytokine secretion. The induction of MOCCI during neuroinflammation triggers the transition of microglia and astrocytes into a neuroprotective state and promotes the resolution of inflammation. Our study therefore provides insights into the relationship between glial cells, mitochondria dysfunction, and neuroinflammation, paving the way for the development of glia-targeted therapies aimed at improving glial metabolic health and function, reducing neurotoxicity and chronic inflammation, and ultimately ameliorating neurological diseases by fostering a restorative CNS milieu.

## Results

### MOCCI expression is upregulated upon inflammation in microglia and astrocytes

MOCCI was discovered in a screen for inflammation-associated peptides that localize to the mitochondria. The C15orf48 gene (AA467197 in mouse), which encodes MOCCI, showed the largest upregulation in transcription and translation after IL-1β treatment in human aortic endothelial cells (22). To see if MOCCI plays a role in neuroinflammation, we sought to determine if MOCCI is upregulated upon inflammatory stimuli in the CNS. As astrocytes and microglia are key players that orchestrate the response to injury and infection in the CNS, we chose to characterize the function of MOCCI in these two cell types.

**Figure 1:**
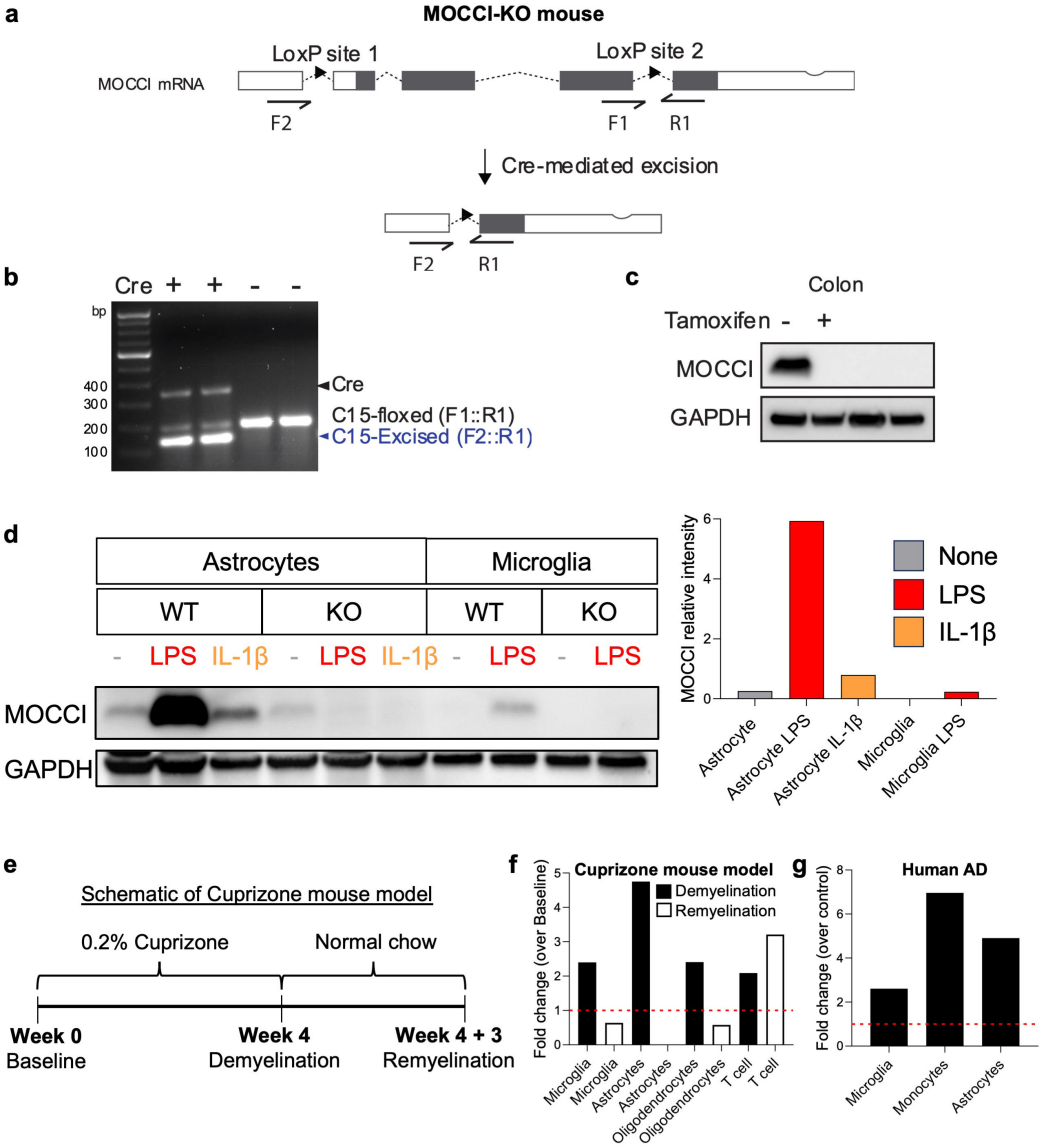
MOCCI expression is triggered by inflammation in astrocytes and microglia. **(a)** Schematic of C15orf48-floxed allele for generating C15orf48-KO mice upon tamoxifen induction. **(b)** PCR gel showing excision of C15orf48 upon tamoxifen induction. **(c)** Western blots of lysed colon tissue derived showing removal of MOCCI protein upon tamoxifen induction. **(d)** Western blots of lysed primary astrocytes and primary microglia derived from C15orf48-WT and C15orf48-KO mice, following LPS and IL-1β treatment. Right graph: Quantification of protein levels of MOCCI relative intensity compared to GAPDH loading control. n=1. **(e)** Schematic of the cuprizone mouse model. **(f)** Quantification of MOCCI transcript (AA467197) in the demyelination and remyelination condition in cuprizone-treated mouse brains (normalized to baseline). Data are averaged and obtained from pseudobulked (by cell type) single-cell RNA-seq of WT cuprizone-treated corpus callosum, at baseline (untreated), 4 weeks (demyelination) and 4 + 3 weeks (remyelination). **(g)** Quantification of MOCCI transcript (C15ORF48) in human AD and control brains (prefrontal cortex). Expression data taken from https://www.synapse.org/Synapse:syn24168322 and https://www.bioinform.cn/SCAD.

To answer the question of whether MOCCI plays a role in the response to inflammation, we generated a C15orf48 knockout by creating a conditional allele crossed to a Rosa-CreERT transgenic, allowing us to induce C15orf48 knockout using tamoxifen (**Figures 1a, b**). We first showed a lack of MOCCI expression upon tamoxifen induction in colon tissue isolated from C15orf48^f/f^;CreERT2 mice, as colon has strong MOCCI expression (**Figure 1c**) (PMID: 12209954). Next, we confirmed a lack of MOCCI expression and induction in astrocytes and microglia derived from C15orf48-WT and C15orf48-KO mice (**Figure 1d**). We observed a faint signal in C15orf48 knockout astrocytes, potentially due to incomplete knockout or residual protein. We then treated primary cultures of mouse astrocytes and microglia with lipopolysaccharide (LPS), and quantified MOCCI expression levels. MOCCI expression is low at baseline but is robustly upregulated upon LPS treatment in both astrocytes and microglia (**Figure 1d**). As MOCCI upregulation was strongest in astrocytes, we also show that IL-1β treatment stimulates MOCCI expression in astrocytes (**Figure 1d**).

To determine if MOCCI expression is upregulated in neuroinflammatory disease models *in vivo*, and to ascertain in which brain cell types it plays a role, we turned to a publicly-available scRNA-seq dataset of the cuprizone model of demyelination and remyelination (23). We compared AA467197 expression in the basal and demyelination/remyelination timepoint (**Figure 1e** schematic), showing that pseudo-bulked profiles of microglia, astrocytes, oligodendrocytes and T cells showed an upregulation of AA467197 at the demyelination timepoint compared to basal conditions (**Figure 1f**). Consistent with our data (**Figure 1d**), AA467197 upregulation was the strongest in astrocytes in the demyelination phase, followed by microglia, oligodendrocytes and T cells, but not in other cell types recovered in that dataset. Interestingly, its expression returns to basal levels in astrocytes, microglia and oligodendrocytes during the remyelination phase but remains consistently high in T cells, likely reflecting their peripheral origin and possibly persistent activation in this disease model.

We next asked if MOCCI is similarly upregulated in humans with an inflammatory CNS disorder such as AD. From a dataset of AD vs control brains (prefrontal cortex) (24), we see that C15orf48 is upregulated in microglia, astrocytes and monocytes (**Figure 1g**). These expression data tell us that MOCCI is upregulated upon inflammation *in vitro* and *in vivo*, as well as in clinically relevant human brain tissue.

### MOCCI drives transcriptional changes in microglia and astrocytes in response to neuroinflammation

**Figure 2:**
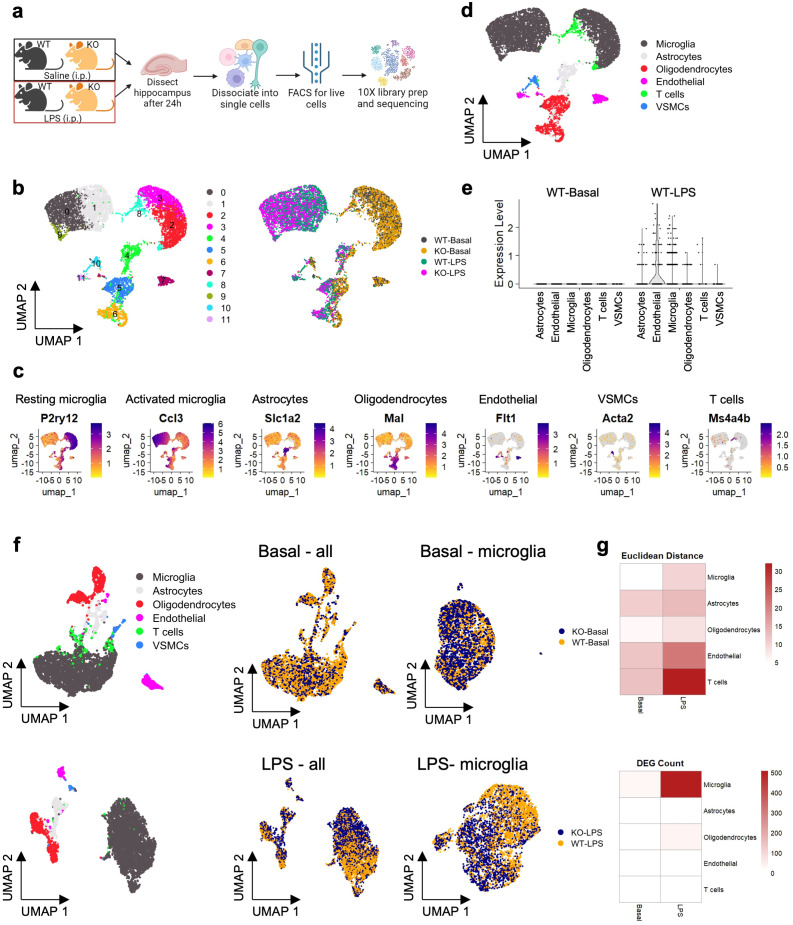
MOCCI expression increases during acute neuroinflammation and modulates glial transcriptional programs. **(a)** Schematic of the experimental design for the scRNA-seq experiment (see Methods). Two hippocampi from one mouse per genotype were used. **(b)** UMAP projection of 10,267 cells, colored by cluster number and genotype/condition. **(c)** UMAP projection colored by selected marker genes for cell type identification. Marker genes used are *P2ry12: resting microglia, Ccl3: activated microglia, Slc1a2: astrocytes, Mal: oligodendrocytes, Flt1: endothelial cells, Acta2: VSMCs, Ms4a4b: T cells*. **(d)** Coarse cell type annotation showing recovery of microglia, astrocytes, oligodendrocytes, endothelial cells, T cells and VSMCs. **(e)** MOCCI transcript (AA467197) is upregulated upon LPS treatment in all recovered cell types. **(f)** Left and middle: UMAP projection of sub-clustered basal-only and LPS-only cells, colored by genotype and cell type, showing more distinct separation between the genotypes during LPS treatment compared to the basal condition. Right: UMAP projection of sub-clustered basal-only and LPS-only microglia. **(g)** Heatmap depicting the Euclidean distance and number of DEGs between C15orf48-WT and C15orf48-KO, showing a sharper distinction between the genotypes in the LPS case compared to basal.

Given that MOCCI expression is most strongly induced in astrocytes and microglia during neuroinflammatory conditions, these glial cell types provide a focused context for investigating how MOCCI modulates inflammation in the brain. We next examined the effect of MOCCI on the activation state of astrocytes and microglia upon LPS stimulation by performing scRNA-seq on the hippocampus of LPS-injected and saline-injected C15orf48-KO vs C15orf48-WT mice (**Figure 2a**, schematic). We focussed on the hippocampus as it is highly responsive to LPS, showing robust glial activation (25, 26). As expected, UMAP clustering of all cells is largely driven by cell type and LPS/basal status, with genotype exerting a smaller effect (**Figure 2b**). Following coarse cell type annotation using a combination of known, canonical marker genes of each cell type (**Figure 2c**), and most highly expressed marker genes of each cluster, we were able to recover the following cell types: microglia, astrocytes, oligodendrocytes, endothelial cells, T cells, and VSMCs (**Figure 2d**). Marker genes used were *P2ry12*: resting microglia, *Ccl3*: activated microglia, *Slc1a2*: astrocytes, *Mal*: oligodendrocytes, *Flt1*: endothelial cells, *Acta2*: VSMCs, *Ms4a4b*: T cells. We next confirmed that the MOCCI transcript, AA467197, is upregulated upon LPS stimulation in WT cells in all cell types recovered (**Figure 2e**), matching our *in vitro* data, as well as in the cuprizone model and human AD brains. Although the proportion of AA467197-positive cells after LPS stimulation is modest, this could reflect stronger response in specific subpopulations, transient induction, post-translational regulation, and/or insufficient scRNA-seq sensitivity or dropout. *In vitro*, astrocytes display stronger LPS-induced MOCCI upregulation than microglia (**Figure 1a**), whereas *in vivo* the upregulation of AA467197 is comparable in both cell types (**Figure 2e**). This possibly reflects either context (*in vitro* vs. *in vivo*) or target (protein vs. transcript) differences. Although our study focuses primarily on microglia and astrocytes, we note that AA467197 upregulation upon LPS stimulation *in vivo* is most pronounced in endothelial cells, which are key regulators of neurovascular inflammation. As this is a global mouse knockout, this raises the possibility that some of the observed glial activation states may be influenced through endothelial–glial crosstalk. Given the close spatial proximity between endothelial cells and glia, such interactions could shape the diversity or intensity of glial activation states (27). Our cell-cell communication analysis revealed increased collagen and fibronectin signaling from C15orf48-KO endothelial cells, compared to the WT endothelium (**Supplementary Figure 2**), highlighting a potential neurovascular mechanism through which MOCCI modulates CNS inflammation.

Because UMAP clusters are largely driven by LPS status, we subclustered basal cells and LPS-treated cells separately. We saw no major differences in the clustering between C15orf48-WT and C15orf48-KO in the basal condition, but a clear separation in the LPS condition (**Figure 2f**, middle). Similarly, we subclustered basal and LPS-treated microglia, and saw broad overlap in the basal condition, and clear separation between C15orf48-WT and C15orf48-KO in the LPS case (**Figure 2f**, right). Next, we quantified the differences between the C15orf48-WT and C15orf48-KO in each recovered cell type, using the number of DEGs and Euclidean distance between the centroids of the two cell populations, to provide a measure of transcriptional difference. We showed a bigger difference between the genotypes following LPS treatment compared to the basal condition (**Figure 2g**). We also showed that microglia had the highest number of DEGs between WT and KO (**Figure 2g**, bottom panel), prompting us to focus on microglia in our downstream analysis. These data point to MOCCI having a role not in the basal, homeostatic environment, but in the response to inflammation.

### MOCCI mediates a shift from a pro-inflammatory transcriptional state to a more pro-resolution transcriptional state in microglia

**Figure 3:**
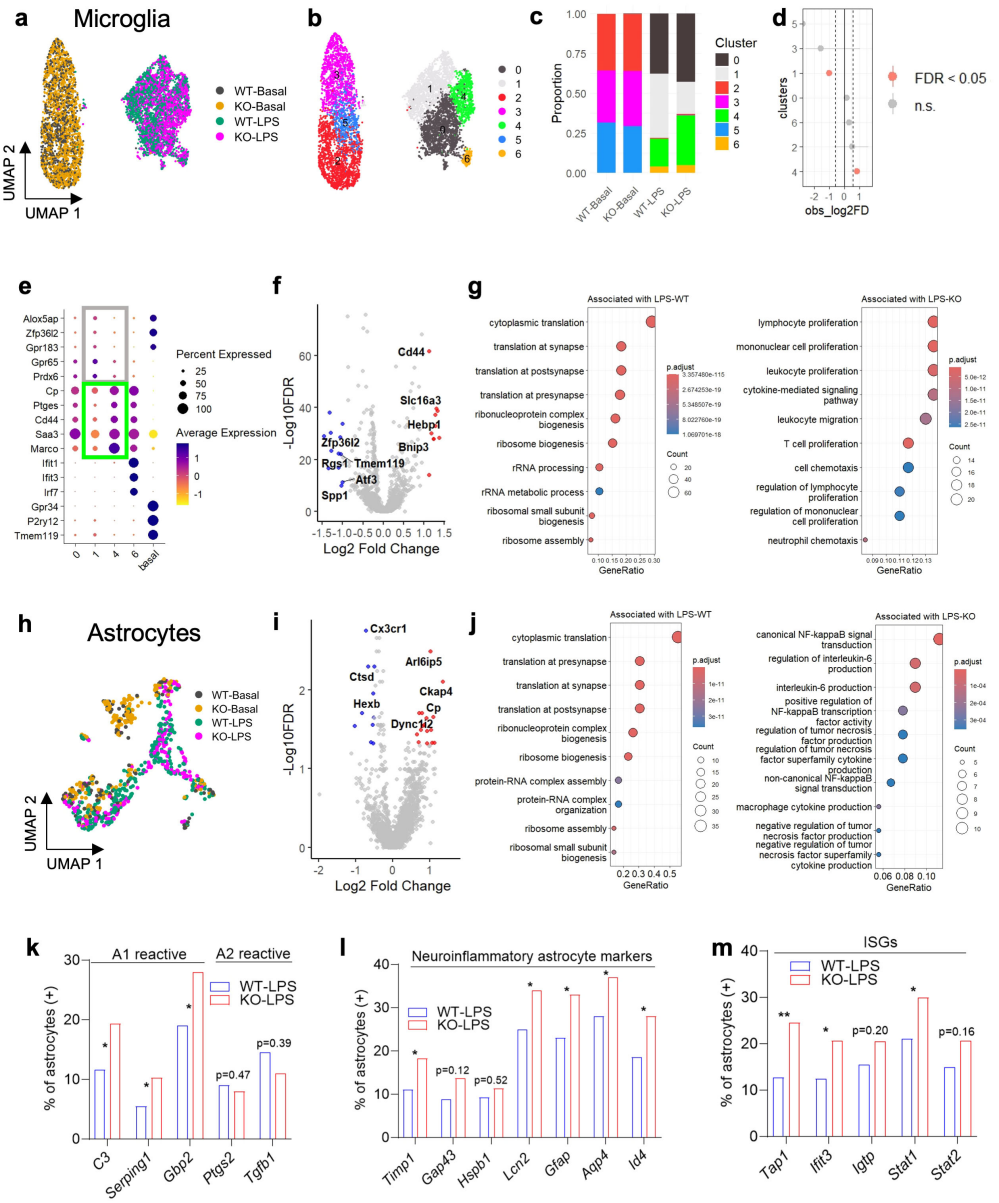
MOCCI modulates glial activation states during neuroinflammation. Microglia-only sub-clustering. UMAP projection of 6948 microglia from the same batch, colored by genotype and treatment condition. **(b)** Microglia-only sub-clustering. UMAP projection of microglia colored by cluster number. WT: Cluster 2, 3, 5. KO: Cluster 0, 1, 4, 6. **(c)** Stacked bar plot showing the proportion of cells from each microglia cluster, for each genotype and condition. **(d)** Statistical significance between WT and KO in the LPS condition assessed using a proportion test, showing a significant difference (FDR < 0.05 and log2FC > 0.58) in clusters 1 and 4. **(e)** Dot Plot showing top marker genes in each LPS cluster and basal. **(f)** Volcano plot showing the DEGs (FDR < 0.05 and abs(log2FC) > 1) between WT and KO in the LPS condition in microglia. **(g)** GSEA of DEGs in **(e)** showing translation related pathways (“Cytoplasmic translation”, “Translation at synapse”) associated with WT, and inflammation-related pathways (“Cytokine mediated signaling pathway”) associated with KO. **(h)** Astrocytes-only sub-clustering. UMAP projection of 797 astrocytes from the same batch, colored by genotype and condition. **(i)** Volcano plot showing the DEGs (FDR < 0.05 and abs(log2FC) > 0.5) between WT and KO in the LPS condition in astrocytes. **(j)** GSEA of DEGs between WT and KO in the LPS condition in astrocytes, showing translation-related pathways (“Cytoplasmic translation”, “Translation at synapse”) associated with WT-LPS astrocytes and IL-6 and NF-κB pathways associated with KO-LPS astrocytes. **(k)** Percentage of astrocytes positive for A1 reactive and A2 reactive astrocyte markers shows that C15orf48-KO brains harbour a higher proportion of A1 reactive astrocytes, but not A2 reactive astrocytes. **(l)** *Timp1, Gap43, Hspb1, Lcn2, Gfap, Aqp4, Id4* are markers of neuroinflammatory astrocytes. C15orf48-KO has a higher proportion expressing these marker genes, suggesting a more inflammatory subtype in C15orf48-KO brains. **(m)** Percentage of astrocytes positive for ISGs show that C15orf48-KO brains harbour a higher proportion of interferon responsive astrocytes. Panels **(k–m)** are descriptive characterizations of astrocyte phenotype within a single scRNA-seq dataset. ns, p >= 0.05; *p < 0.05; **p < 0.01.

We next sub-clustered microglia only and saw basal and LPS-treated microglia being separated into two main clusters: clusters 2, 3, 5 for basal cells and clusters 0, 1, 4, 6 for LPS-treated cells (**Figures 3a–c**). We then dived deeper into the LPS clusters, to determine how the cluster proportions shift, comparing C15orf48-WT and C15orf48-KO (**Figure 3c**). A proportion test showed a significant difference between the genotypes for cluster 1 and 4 (**Figure 3d**). C15orf48-KO causes a shift from clusters 1 to 4, suggesting that MOCCI affects the activation state of microglia upon LPS-induced inflammation. We next wanted to determine what clusters 1 and 4 represent. Thus, we looked at top marker genes of each cluster, grouping the basal clusters 2, 3, 5, as “basal”. As expected, the basal cluster is marked by resting microglia markers such as *P2ry12, Tmem119, Gpr34*. Cluster 6 is an interferon-related microglia cluster, marked by interferon signaling genes such as *Irf7, Ifit1, Ifit3*. Cluster 4 is marked by *Marco*, a gene known to be up-regulated during LPS stimulation and induces cytoskeleton modifications in dendritic cells (28); *Saa3*, which stimulates the expression of the NLRP3 inflammasome (29); *Cd44*, which has been implicated in inflammation associated with ALS and neuronal injuries (30); *Ptges*, which accelerates neurotoxicity (31); and *Cp* which stimulates the expression of proinflammatory factors in microglia (32). In contrast, cluster 1 is marked by *Prdx6*, which mediates penetration and clearance of Aβ plaques (33); *Gpr65*, which inhibits glial scarring and is neuroprotective (34), and *Zfp36l2*, which serves as an anti-inflammatory modulator (35) (**Figure 3e**). This suggests that cluster 4 could represent a pro-inflammatory activation state, while cluster 1 microglia represent a less inflammatory, pro-resolution activation state. The observed changes in the KO (**Figure 3d**) suggest that MOCCI-deficient microglia are linked to more pro-inflammatory transcriptional changes upon LPS stimulation.

We next looked at differentially expressed genes (DEGs) in microglia following LPS treatment. C15orf48-KO microglia upregulate *Bnip3*, a mitochondrial protein known to be involved in mitophagy (36); and *Slc16a3*, which encodes a monocarboxylate transporter involved in lactate metabolism (37). Among down-regulated genes are *Rgs1*, that induces inflammation via the NF-κB and p38 pathways (38); *Atf3*, a transcription factor that mediates oxidative stress and the immune response (39); *Spp1*, a marker of activated microglia that is induced in many neuropathological conditions; and *Tmem119*, typically associated with resting microglia (**Figure 3f**). This suggests that there are distinct activation states in microglia that do not conform to the resting-activated spectrum, but could have different activation states over time, or under different conditions. GSEA analysis of DEGs showed translation related processes being down-regulated and cytokine-related and chemotaxis and proliferation pathways being up-regulated in C15orf48-KO microglia (**Figure 3g**). We also measured the expression of key M1 pro-inflammatory and M2 anti-inflammatory markers in microglia. Upon LPS stimulation, MOCCI-KO microglia exhibited significantly elevated expression of M1 markers (*Il1b, Ccl3, Ccl4*) compared to WT (**Supplementary Figure 1a**). Importantly, in general, in genes where LPS induced upregulation relative to basal conditions, MOCCI-KO showed a higher expression compared to MOCCI-WT. Conversely, homeostatic microglial markers (*Cx3cr1, Tmem119, P2ry12*) were markedly downregulated in the LPS condition, and this downregulation was more pronounced in KO compared to WT microglia (**Supplementary Figure 1b**). Taken together with evidence showing that mitochondrial activity sustains neuroinflammation (40) and that MOCCI plays a role in tempering the immune response (22), our data suggest that MOCCI could act as a molecular brake, whereby loss of MOCCI results in pro-inflammatory-like transcriptional changes and reduced protein synthesis, potentially reflecting cellular stress or an energy-conserving adaptive response (41, 42).

### C15orf48-KO brains exhibit a higher proportion of A1 reactive and interferon-response astrocytes

We next turned our attention towards astrocytes. UMAP projection of astrocyte sub-clustering shows a more subtle differentiation between basal and LPS cells, compared to that seen in microglia (**Figure 3f**). We next looked at DEGs between C15orf48-WT and C15orf48-KO cells in the LPS condition. Loss of MOCCI in astrocytes caused a distinct transcriptional shift characterized by the upregulation of genes such as *Cp, Dync1i2, Ckap4*, and *Arl6ip5*, which are associated with an oxidative stress response, ER and cytoskeleton remodeling, and glutamate homeostasis (43–46). We also observed down-regulation of *Cx3cr1, Ctsd*, and *Hexb* in C15orf48-KO astrocytes, genes that are involved in immune signaling and lysosomal function (47–49) (**Figure 3i**). This suggests that loss of MOCCI drives astrocytes towards a reactive phenotype marked by increased cellular stress management and organelle dynamics, while diminishing immunomodulatory and lysosomal degradative capacity. GSEA analysis shows translation-related pathways associated with C15orf48-WT astrocytes, similar to that observed in microglia, and Il-6 and NF-κB pathways associated with C15orf48-KO (**Figure 3j**). This suggests that loss of MOCCI shifts astrocytes towards a pro-inflammatory-like transcriptional profile, consistent with its role in suppressing cytokine secretion in some cell types (22). However, as this was observed *in vivo*, it is unknown whether the observed transcriptional changes reflect a cell-autonomous effect of MOCCI loss in astrocytes or are influenced by MOCCI deficiency in other cell types in the brain, such as the endothelium.

Going back to the classical model of A1 and A2 reactive astrocytes, we saw that the proportion of astrocytes expressing canonical A1 markers (*C3, Serping1, Gbp2*) are higher in C15orf48-KO brains compared to WT, while the proportion of astrocytes expressing canonical A2 markers (*Ptgs2, Tfgb1*) was unchanged (**Figure 3k**). A1 astrocytes are less phagocytic, less neurotrophic, secrete more pro-inflammatory factors, and are enriched in neurodegenerative brains such as in AD, where they can contribute to chronic neuroinflammation and neuronal death (50). Because A1 astrocytes can be induced by pro-inflammatory cytokines released from activated microglia, the A1 astrocyte enrichment may be driven, at least in part, by C15orf48-KO microglia. We next wanted to see how our observed astrocytes states correlate with previously published and defined astrocyte clusters/states (51). Upon C15orf48-KO, we observed an increase in the proportion of astrocytes expressing *Timp1, Gap43, Hspb1, Lcn2, Gfap, Id3, Id4*, markers that define “cluster 4 neuroinflammatory astrocyte subtype” in the published report by Hasal et al. (**Figure 3l**). C15orf48-KO mice have a greater proportion of neuroinflammatory astrocytes compared to C15orf48-WT upon LPS stimulation, which could suggest that MOCCI induction serves to restrain the inflammatory response to LPS. We also observed an increase in the proportion of astrocytes expressing interferon-response genes (**Figure 3m**), which we did not observe in microglia. This implies MOCCI may exert different effects on different glial populations. While MOCCI deficiency pushes microglia to a pro-inflammatory activation state, astrocytes undergo a subtler remodeling, marked by stress-adapted, A1-like, and interferon-responsive signatures. This is perhaps due to differences in metabolic and cellular demands, or that microglia and astrocytes are differentially affected by an inflammatory milieu.

### MOCCI modulates cell–cell communication networks in the brain

**Figure 4:**
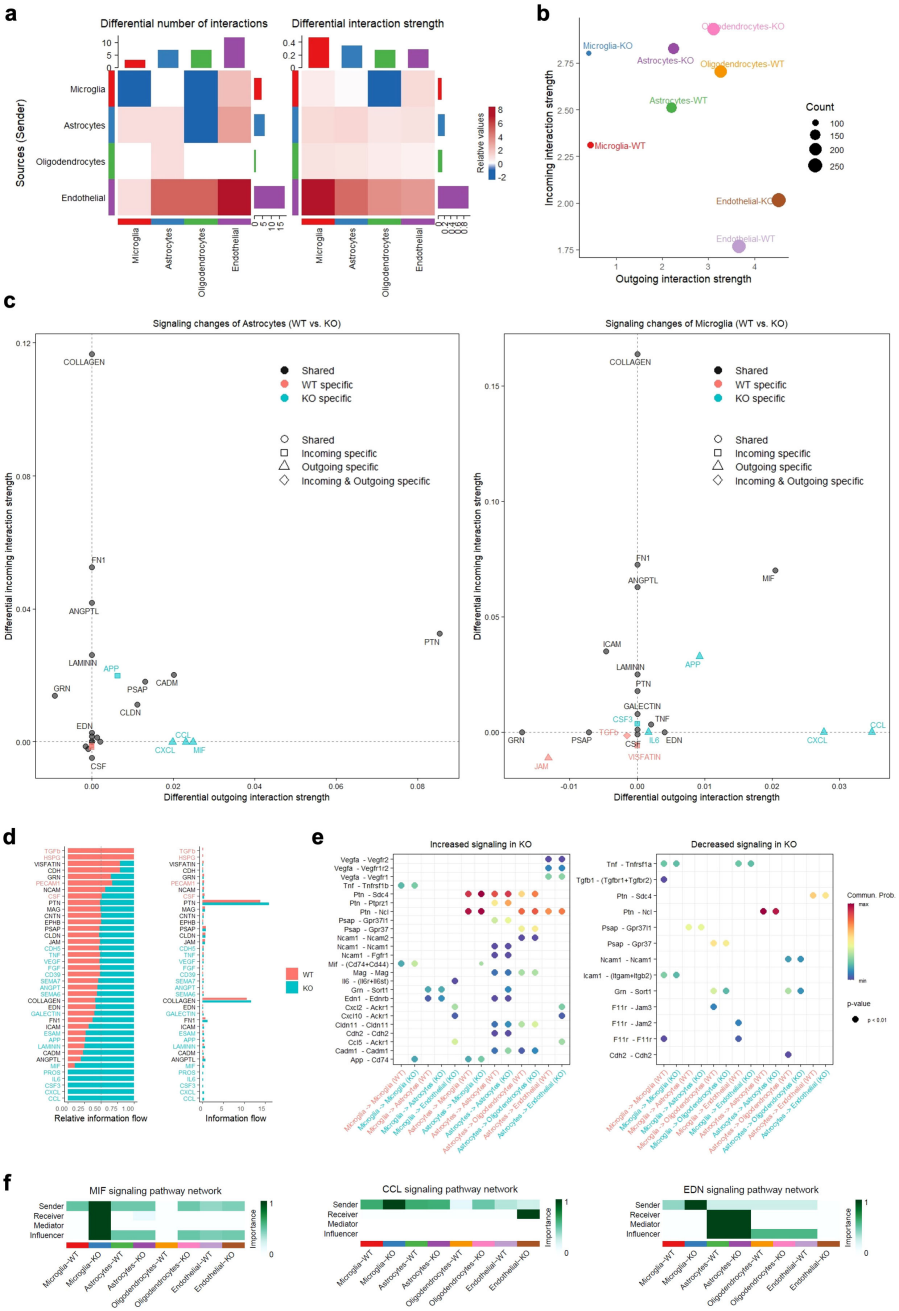
Loss of MOCCI disrupts cellular crosstalk during neuroinflammation. **(a)** Heatmap showing changes in interaction number and interaction strength. Red: increased in KO. Blue: increased in WT. **(b)** Scatter plot showing incoming and outgoing overall interaction strengths for each cell type/genotype. **(c)** Scatter plot showing incoming and outgoing signaling pathways, as well as differential signaling pathway strengths between C15orf48-WT and C15orf48-KO in the LPS condition, in microglia (left) and astrocyte (right). **(d)** Comparison of overall information flow across signaling pathways between C15orf48-WT and C15orf48-KO brains. Left: Stacked bar plot showing the relative information flow. Right: Bar graph of absolute information flow per signaling pathway. Signaling pathways are ranked based on the difference in information flow between conditions. Pathways highlighted in red are significantly enriched in WT brains, while those in green are enriched in KO brains. **(e)** Comparison of communication probabilities mediated by individual ligand–receptor pairs focusing on signaling pathways where microglia or astrocytes are the source cells. This highlights dysfunctional signaling changes caused by loss of MOCCI. Left: Ligand–receptor interactions with increased communication probability in C15orf48-KO compared to C15orf48-WT. Right: Ligand–receptor interactions with increased communication probability in C15orf48-WT compared to C15orf48-KO. **(f)** Heatmap showing each cell group’s contributions to individual signaling pathways: MIF, CCL, EDN, showing increased contribution in C15orf48-KO microglia compared to C15orf48-WT microglia as a ligand source in these three pathways.

Given the *in vivo*, multicellular context of our observations, we wanted to explore how MOCCI influences intercellular communication in the brain. We turned to CellChat (52) to map changes in ligand-receptor signaling pathways between the different cell types. First, we showed differential number and strength of ligand-receptor interactions (**Figure 4a**), with an overall increase in signaling in C15orf48-KO compared to WT (**Figure 4b**). CellChat analysis revealed that the differences between incoming signals was greater than that of outgoing signals between KO and WT, suggesting that C15orf48-KO glia exhibit higher expression of signaling receptors, which could reflect an altered responsiveness to cues from the surrounding cellular environment. Next, we looked at specific pathway changes in astrocytes and microglia (**Figures 4c–f**). We observed an increase in the MIF (microglia and astrocytes as senders, microglia as receivers) signaling pathway (**Figure 4f**), suggesting an increased pro-inflammatory communication profile (53). In addition, we observed an increase in EDN pathway signaling (54), where microglia are the senders and astrocytes the receivers (**Figure 4f**). Because these pathways are known to mediate cytokine signaling and microglia activation, loss of MOCCI may amplify neuroinflammatory signaling cascades, consistent with its role in attenuating overactive inflammatory signaling. Interestingly, endothelial cells in C15orf48-KO brains show an upregulation of *Ackr1* (**Figure 4e**, left), a chemokine “sink”, suggesting a possible compensatory loop where overproduction of cytokines and chemokines in the MOCCI-deficient brain by KO astrocytes and microglia drives *Ackr1* expression on endothelial cells to sequester/clear cytokines and chemokines (55). EDN signaling, shown to be increased in C15orf48-KO microglia (**Figure 4e**, left), is also known to inhibit remyelination (56). Differential signaling pathways where either microglia or astrocytes are senders are depicted in **Figure 4e**, and all differential signaling pathways are shown in **Supplementary Figure 2**.

### MOCCI is required for efficient migration and phagocytosis in microglia and astrocytes

In response to inflammatory triggers, microglia and astrocytes demonstrate remarkable abilities in phagocytosis, migration and cytokine secretion. They migrate swiftly to affected areas of the brain and engage in phagocytosis to clear debris and pathogens, promoting tissue repair. Following our observation that MOCCI affects the state of microglia and astrocytes transcriptomically, we wanted to see if MOCCI is required for their roles in cellular phagocytosis, migration and cytokine production, *in vitro* and in isolation.

**Figure 5:**
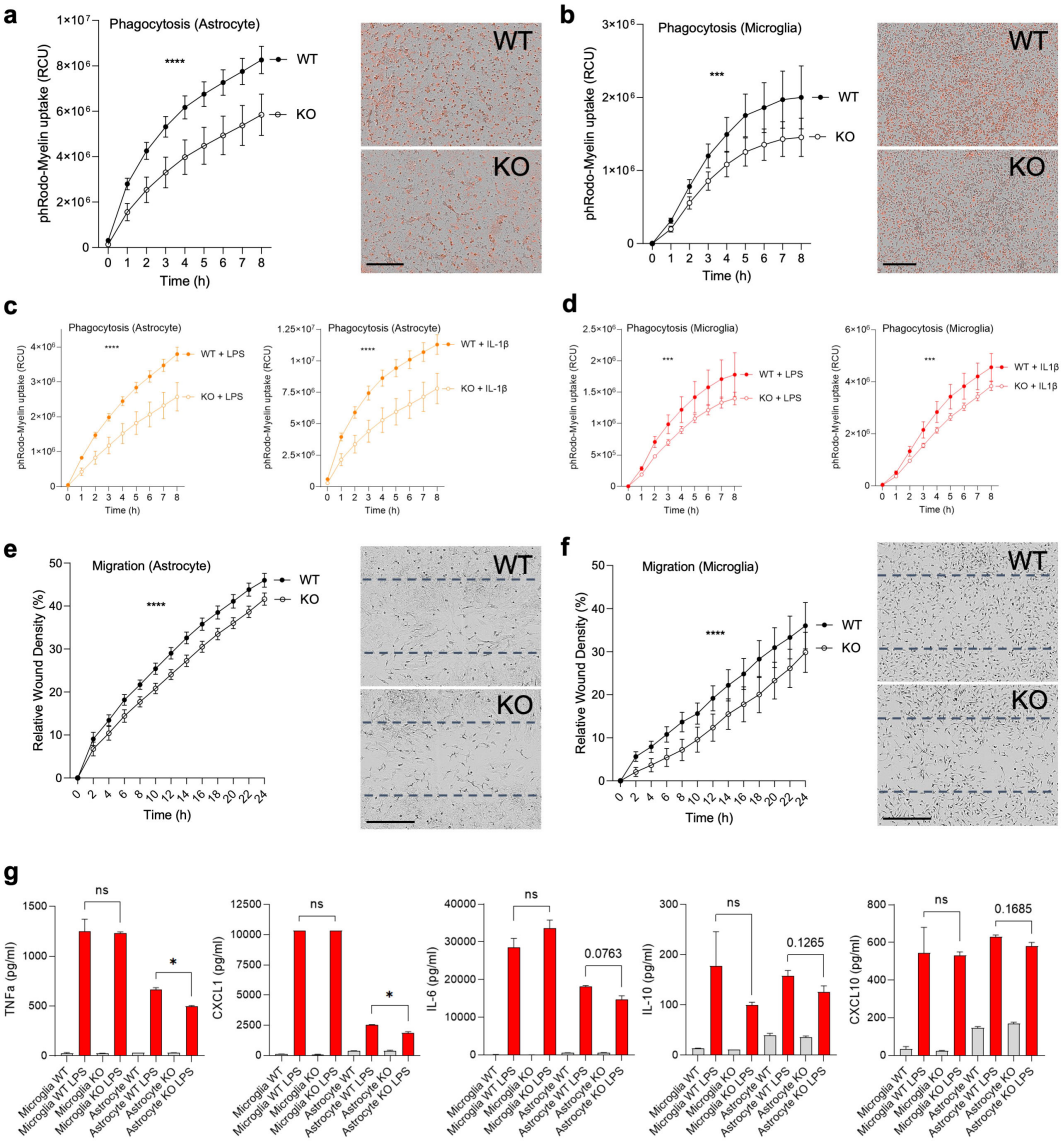
MOCCI regulates phagocytosis, migration and cytokine secretion in glial cells *in vitro*. **(a)** Left: Uptake of phRodo-labeled myelin debris in astrocytes is significantly lower in C15orf48-KO compared to WT (p-val <0.0001, n = 6 experiments, with 2–3 animals per genotype per experiment). Data were also analyzed by two-way ANOVA with genotype (WT vs KO) and treatment (baseline, LPS, IL-1β) as factors. A significant main effect of genotype (F(1,30) = 14.68, p = 0.0006), with overall lower values in KO compared to WT. Sidak’s multiple comparisons test revealed a significant WT–KO difference under IL-1β stimulation (adjusted p = 0.0086), whereas genotype-dependent differences at baseline (adjusted p = 0.093) and with LPS treatment (adjusted p = 0.599) did not reach significance. Right: Representative images of astrocytes after 8 h incubation with phRodo-labeled myelin shows reduced phagocytic capabilities of C15orf48-KO astrocytes compared to WT. Scale bar: 300 μm. **(b)** Left: Uptake of phRodo-labeled myelin debris in microglia is significantly lower in C15orf48-KO compared to WT (p-val <0.0007, n = 2 experiments, with 2–3 animals per genotype per experiment). Data were also analyzed by two-way ANOVA with genotype (WT vs KO) and treatment (baseline, LPS, IL-1β) as factors. The main effect of genotype did not reach, but trends towards significance (F(1,6) = 5.546, p = 0.0567). Right: Representative images of microglia after 8 h incubation with phRodo-labeled myelin shows reduced phagocytic capabilities of C15orf48-KO microglia compared to WT. Scale bar: 300 μm. **(c)** Uptake of phRodo-labeled myelin debris upon LPS or IL-1β stimulation in astrocytes is significantly lower in C15orf48-KO compared to WT (p-val <0.0001, n = 6 experiments, with 2–3 animals per genotype per experiment). **(d)** Uptake of phRodo-labeled myelin debris upon LPS or IL-1β stimulation in microglia is significantly lower in C15orf48-KO compared to WT (p-val <0.0001, n = 2 experiments, with 2–3 animals per genotype per experiment). **(e)** Left: Cellular migration, measured by relative wound density (density of cells inside scratch wound, relative to density of cells outside scratch wound), is inhibited in C15orf48-KO astrocytes compared to WT (p-val <0.0001, n = 6 experiments, with 2–3 animals per genotype per experiment). Right: Representative images of scratch wound image after 24 h. Area between dotted lines delineate scratch wound area, showing reduced wound closure in C15orf48-KO compared to WT. Scale bar: 300 μm. **(f)** Left: Cellular migration, measured by relative wound density (which is the density of cells inside scratch wound, relative to density of cells outside scratch wound), is inhibited in C15orf48-KO microglia compared to WT (p-val <0.0001, n = 2 experiments, with 2–3 animals per genotype per experiment). Right: Representative images of scratch wound image after 24 h. Area between dotted lines delineate scratch wound area, showing reduced wound closure in C15orf48-KO compared to WT. Scale bar: 300 μm. **(g)** TNFα, IL-6, CXCL1, IL-10, CXCL10 levels in astrocyte and microglia conditioned media following LPS stimulus, showing a muted cytokine secretion response in MOCCI-KO astrocytes compared to WT (n = 2 experiments, with 2–3 animals per genotype per experiment). MOCCI-KO microglia did not show a similar inhibition in cytokine secretion. Data are represented as mean ± SEM. Statistical significance displayed was determined using a two-tailed unpaired t-test (ns, p ≥ 0.05; *, p < 0.05; **, p < 0.01; ***, p < 0.001; ****, p < 0.0001).

To directly compare phagocytic uptake capacity of C15orf48-WT and C15orf48-KO *in vitro*, we used phRodo-labeled myelin, which fluoresces upon entry into acidic intracellular compartments. Myelin debris itself serves as an inflammatory trigger to stimulate MOCCI production. The observed difference in myelin phagocytosis between MOCCI-WT and MOCCI-KO cells (**Figures 5a, b**) provides indirect evidence that MOCCI is induced upon myelin debris exposure, despite the fact that we did not directly measure MOCCI expression levels in response to myelin addition. We note this as an important limitation and direction for follow-up work, where direct measurement of MOCCI expression levels in response to myelin debris (by qPCR or Western blot) would be useful. Using this assay, C15orf48-KO microglia and astrocytes showed reduced uptake of phRodo-myelin (**Figures 5a, b**). Stimulation with IL-1β and LPS also shows a similar decrease of phRodo-myelin uptake in C15orf48-KO primary astrocytes and microglia (**Figures 5c, d**). To assess migration, we used a scratch-wound assay and quantified wound closure. Similar to phagocytosis, we observed a decrease in wound closure in C15orf48-KO astrocytes and microglia compared to WT (**Figures 5e, f**). Representative images of the cultured astrocytes and microglia are shown (**Figures 5a, b, e, f**), and both cell density and cellular morphology are comparable between WT and KO, arguing against differential cell loss as the basis for reduced myelin uptake and migration. Using a luminex assay on conditioned media, we see a muted cytokine secretion upon LPS stimulation in astrocytes, but not in microglia (**Figure 5g**). Since we observed an increase in cytokine expression in MOCCI-KO microglia *in vivo*, it is possible that culture conditions do not fully recapitulate the *in vivo*, physiological system, and the lack of other cells/stimulants in its environment. An alternative interpretation of the reduced cytokine secretion in MOCCI-KO astrocytes is that it reflects a general attenuation of translational capacity, consistent with the downregulation of ribosomal and translation-related transcripts observed in KO astrocytes (**Figure 3**). Overall, our results show that MOCCI is required for appropriate glial activation and subsequent morphological and functional changes, specifically phagocytosis and migration.

### MOCCI deficiency alters cuprizone-mediated demyelination and remyelination

Since MOCCI affects glial activation, a cellular process critical in orchestrating neuroinflammation and the progression of neurodegeneration, we hypothesized that MOCCI could play a role in slowing neurodegenerative disease progression. We therefore turned to the cuprizone model to assess the role of MOCCI in demyelination and remyelination. Cuprizone exposure leads to oligodendrocyte death and therefore demyelination. Withdrawal of cuprizone leads to spontaneous remyelination in WT animals. Important cellular processes following demyelination are the migration of microglia and astrocytes into the demyelinated lesion, followed by phagocytosis of cellular debris. The clearance of cellular debris underlies recovery and remyelination (57). We fed mice 0.2% cuprizone feed for 4 weeks to cause demyelination in the corpus callosum (4 week timepoint), followed by 3 weeks of normal feed to allow remyelination (4 + 3 week timepoint) (**Figure 1b**, schematic). AA467197 is upregulated upon demyelination compared to expression at baseline (**Figure 1c**).

**Figure 6:**
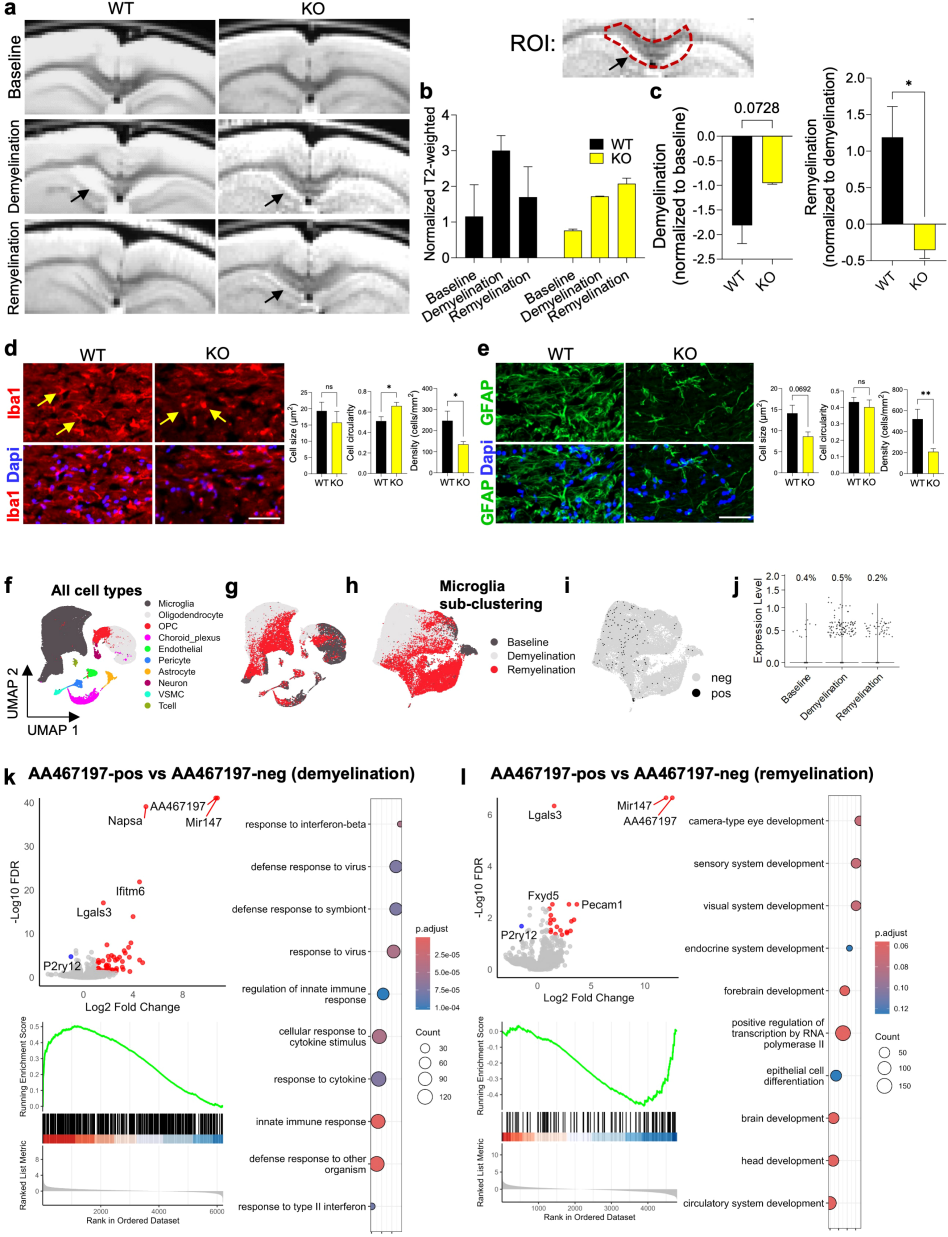
MOCCI mediates glial responses *in vivo* during cuprizone-induced demyelination and remyelination. **(a)** T2-weighted images of the corpus callosum at baseline, 4 weeks demyelination, and 4 + 3 weeks remyelination timepoints. Blue arrows point to white cuprizone-induced demyelinated lesions, showing reduced demyelination, and reduced recovery of demyelinated lesions in C15orf48-KO compared to WT. n = 2 mice per genotype. A representative region of interest (ROI) for quantification is shown on the right (red dashed lines). **(b)** Quantification of normalized signal intensities in the ROI for each timepoint and genotype, showing impaired demyelination and remyelination in C15orf48-KO mice compared to WT. **(c)** Left: Quantification of demyelination (signal intensity baseline – signal intensity of demyelination), showing a trend towards significance between C15orf48-WT and C15orf48-KO (p-val = 0.0728). The more negative the values, the greater the demyelination. Right: Quantification of remyelination (signal intensity of demyelination – signal intensity of remyelination) showing a significant difference between C15orf48-WT and C15orf48-KO (p-val = 0.0356).More positive values represent greater remyelination. **(d)** Iba1 antibody staining for microglia in the lesion, at the remyelination timepoint, showing a more rounded morphology in C15orf48-KO compared to WT (yellow arrows). Left: Quantification of cell size, circularity and densities. Scale bar: 50 μm. **(e)** GFAP antibody staining for astrocytes in the lesion, at the remyelination timepoint, showing reduced astrocyte density in C15orf48-KO compared to WT. Scale bar: 50 μm. Left: Quantification of cell size, circularity and densities. **(f, g)** UMAP projection from a publicly available dataset (23), of WT corpus callosum tissue from cuprizone-treated mice, colored by cell type **(f)** and time point **(g, h)** Microglia-only sub-clustering of dataset from **(f, g)** colored by time point. **(i)** Microglia-only sub-clustering showing AA467197 (MOCCI)-positive/negative cells. **(j)** Expression levels of AA467197 (MOCCI) in microglia. **(k, l)** Volcano plot, GSEA plot (running enrichment score shown as green line with ranked gene list), and dot plot showing top GO terms comparing MOCCI-positive vs. MOCCI-negative microglia, for the demyelination **(k)** and remyelination **(l)** timepoints, identifying molecular programs associated with MOCCI expression in the cuprizone disease context. Data are represented as mean ± SEM. Statistical significance was determined using a two-tailed unpaired t-test. ns, p ≥ 0.05, *p < 0.05, **p < 0.01.

We used MRI to compare the cuprizone-induced lesion in C15orf48-WT and C15orf48-KO brains (n = 2 mice per group). Unexpectedly, during demyelination, at the 4 week timepoint, lesions in C15orf48-KO brains were smaller than WT lesions (**Figures 6a–c**). This may reflect differences in debris clearance dynamics of dead or dying oligodendrocytes, a process in which glial activation plays a central role. Importantly, C15orf48-KO animals did not recover as well as C15orf48-WT animals following the withdrawal of cuprizone (**Figures 6a–c**), with lesions persisting at 4 + 3 weeks only in the KO. Because MOCCI deficiency in astrocytes and microglia inhibits phagocytosis and migration (**Figures 5a–f**), it is possible that MOCCI deficiency could affect efficient clearance of cellular debris during demyelination and remyelination. Given the relatively small sample sizes in these analyses, further studies will be required to define the underlying mechanisms. Clearance is important because presence of debris inhibits remyelination and recovery (23, 57). We next looked at the morphology of microglia and astrocytes at a 4 + 6 week timepoint (6 weeks after recovery) by histology. Microglia in both genotypes do not have a stereotypical ramified morphology suggestive of resting microglia, and microglial morphology appears smaller and more rounded in C15orf48-KO compared to WT, characteristic of a more activated state, rather than the ramified morphology typical of resolution (**Figure 6d**). This is in line with the incomplete remyelination observed, suggesting that sustained inflammation in the absence of MOCCI may inhibit the transition to a restorative environment necessary for remyelination. Astrocytes have also been shown to be present in high density in remyelinated lesions following cuprizone treatment (23). This is due to their roles in providing neurotrophic and structural support to neurons and oligodendrocytes following remyelination. C15orf48-KO animals exhibit notably reduced astrocyte density in the lesion compared to WT animals (**Figure 6e**). This observation is in line with MOCCI being required for proper astrocyte activation and migration. The observed reduced astrocyte density could contribute to impaired remyelination and tissue repair mechanisms.

We next studied MOCCI expression in a publicly available scRNA-seq dataset of the cuprizone model (23) consisting of three timepoints of baseline, demyelination (4 weeks) and remyelination (4 + 3 weeks) (**Figures 6f, g**; **Supplementary Figure 3**). Focussing on microglia, we show MOCCI expression (AA467197) is the highest in the demyelination timepoint (**Figures 6i, j**). To investigate the role of MOCCI in the microglial responses to injury, we compared MOCCI-positive and MOCCI-negative microglia at both the demyelination and remyelination timepoints. We first confirmed that mir147 expression is tightly and positively correlated with MOCCI (22). Notably, *P2ry12*, a marker of homeostatic microglia, was downregulated in MOCCI-positive cells and *Lgals3*, a marker of activated microglia was upregulated in MOCCI-positive cells, suggesting that MOCCI expression is associated with a more activated microglial profile. This may seem contradictory to the more pro-inflammatory status in C15orf48-KO glia in LPS-treated brains but reflects its upregulation in response to pro-inflammatory signals, as part of a negative feedback loop. Consistent with this model, C15orf48-KO animals exhibited increased expression of inflammatory markers, reflecting its function as a “brake” on inflammation. Whereas in WT animals, MOCCI-positive microglia showed reduced inflammatory gene expression compared to their MOCCI-negative counterparts (**Supplementary Figure 4a**), reflecting the effect of inflammation on MOCCI expression. GSEA analysis of DEGs associated with MOCCI expression shows immune response pathways (“response to interferon beta”, “defense response to virus”) at the demyelination timepoint, and development pathways (“brain development”, “eye development”) at the remyelination timepoint (**Figures 6k, l**). This suggests that MOCCI expression is correlated with glia activation during demyelination, and as remyelination progresses, MOCCI expression is correlated with a restorative phenotype, supporting tissue repair and development. MOCCI may act as a temporal marker of microglial function, linking immune restraint to the onset of repair programs.

## Discussion

In this study, we present evidence that the mitochondrial microprotein MOCCI regulates neuroinflammation by modulating glial activation states. MOCCI is induced in response to inflammatory stimuli in both microglia and astrocytes (**Figure 1**), and facilitates a shift toward a pro-resolution activation state in glial cells upon LPS stimulation (**Figure 3**), thereby playing a critical role in the cellular response to inflammation. We also showed that MOCCI acts as a critical modulator of glial crosstalk during LPS-induced neuroinflammation (**Figure 4**), restraining excessive pro-inflammatory cytokine signaling between microglia, astrocytes, and other cell types in the brain. In the presence of neuroinflammation without MOCCI, enhanced MIF and EDN signaling could contribute to excessive glial activation and a neuroinflammatory environment that may hinder recovery processes. Additionally, MOCCI is essential for appropriate glial responses during cuprizone-mediated demyelination and subsequent remyelination (**Figure 6**). While its role in remyelination is consistent with the observed phagocytic and migratory defects in MOCCI KO microglia and astrocytes *in vitro* (**Figure 5**), we were surprised that MOCCI deficiency reduced the extent of cuprizone-induced demyelination. This could reflect impaired microglial activation and phagocytic function, limiting their ability to effectively clear dying oligodendrocytes (**Figure 6k**).

While we determined the genetic mechanism linking MOCCI to functional glial activation states, the molecular mechanism behind this is currently unknown and requires further investigation. Three recent studies reported the independent generation of MOCCI knockout mice – named iTie (58), Nmes1 (59), and AA467197 (60), and reported its function in regulating innate immune responses. Xia and colleagues recently reported that AA467197 binds to GSDMD to inhibit its activation downstream of NLRP6, and that AA467197 knockout mice had increased susceptibility to both viral and bacterial infections due to an increase in microbial-triggered pyroptotic cell death. In a similar vein, the same group reported that because the MOCCI (C15ORF48/AA467197/iTie) transcript is expressed very highly and constitutively in enterocytes, iTie knockout led to body weight loss in mice, accompanied by reduced length of the small intestines and intestinal villi, and an increased susceptibility to both commensal and pathogenic intestinal bacteria. It is hence proposed that iTie binds to NLRP6 to prevent its activation. iTie deficiency leads to hyperactivation of inflammasomes in enterocytes, compromising intestinal integrity. These findings suggest that MOCCI may act as a negative regulator of inflammasome activation. It is thus plausible that MOCCI modulates glial activation in the brain through a similar mechanism. We showed that *Saa3* is upregulated in C15orf48-KO microglia. *Saa3* is a well-known activator of the Nlrp3 inflammasome (61), suggesting that MOCCI could play a role in restraining inflammasome activity in the brain. Transcriptomically, microglia in C15orf48-KO LPS brains had lower expression of *Gsdmd*, whereas MOCCI-expressing cells in 4-week cuprizone mice exhibited higher *Gsdmd* expression, possibly reflecting context-dependent and protein-level regulation of Gsdmd (**Supplementary Figure 4b**). Future work will be necessary to determine whether MOCCI directly regulates inflammasome components in the CNS, and which cell types mediate this effect. Recently, Clarke and colleagues reported that MOCCI downregulates NDUFA4 to promote the expansion of CXCL9^+^ tumor-associated macrophages via activation of the cGAS–STING pathway (60). Consistent with this, we observed that both C15orf48-KO microglia in LPS-treated brains and non–MOCCI-expressing microglia in 4-week cuprizone brains exhibited reduced cGas transcript levels alongside a modest increase in *Ndufa4* transcript levels (**Supplementary Figure 4c**). Taken together, our results extend the role of MOCCI beyond the periphery, identifying it as a regulator of innate immune signaling in microglia.

A limitation of this study is the use of a full-animal, whole-transcript knockout, where we did not separate MOCCI’s protein-coding function from the effects of miR-147 (22), and were unable to study cell-type–specific roles from the confounding influence of intercellular crosstalk in the brain. In particular, the elevated microglial MIF observed in MOCCI-KO animals (**Figure 4**) could reflect a cell-autonomous role for microglial-derived MOCCI in restraining MIF production, or an indirect effect of MOCCI loss in astrocytes, endothelial cells, or other neighboring cell types. A second limitation is the lack of temporal resolution, making it challenging to distinguish short-term versus long-term effects of MOCCI loss and to dissect primary immune responses from changes driven by feedback loops.

MOCCI is encoded within the miR-147 precursor, and our knockout strategy disrupts both the protein-coding sequence and miR-147. In our previous study (22), we characterized MOCCI (C15ORF48) as a mitochondrial small open reading frame-encoded peptide that modulates inflammation both through its protein function, by replacing NDUFA4 in Complex IV, and also via miR-147, which targets NDUFA4 mRNA. This current study does not disentangle the contributions of MOCCI and miR-147 to microglial modulation. Future work using selective genetic targeting will be required to define their individual and likely synergistic roles in regulating glial activation.

Another limitation of this study is the lack of a tamoxifen-only control. However, all *in vivo* comparisons were made between tamoxifen-treated Cre+ (MOCCI-KO) and tamoxifen-treated Cre– littermate controls, such that any off-target effects of tamoxifen on glia are present in both groups and cannot account for the observed differences.

One of our key observations is the differential effect of MOCCI on microglial and astrocytic activation states, suggesting that MOCCI regulates glial cells in a cell-type or context-dependent manner. In the absence of MOCCI, microglia show an upregulation of chemotaxis and proliferation related genes (**Figure 3g**), while in astrocytes, C15orf48-KO upregulate IL-6 and NF-κB pathways (**Figure 3j**). This divergence could be attributed to differences in oxidative stress handling between the two cell types. While not well-studied, some reports suggest that astrocytes have more robust antioxidant defenses due to their role in oxidative stress regulation in the CNS (62). Microglia rely heavily on mitochondrial metabolism and may be more vulnerable to changes in ROS levels, leading to increased production of chemokines and proliferation in the absence of MOCCI. In contrast, astrocytes, with their more robust antioxidant systems, overactivate IL-6 and NF-κB transcriptional programs in the absence of MOCCI during inflammation. It is also likely, given the extensive crosstalk between cell types in the brain, that MOCCI deficiency in one cell type drives changes in another (**Figure 4**; **Supplementary Figure 2**). For instance, MIF and EDN signaling from microglia to astrocytes is increased in C15orf48-KO (**Figure 4f**). These changes point to a coordinated, multicellular response to neuroinflammation shaped by MOCCI. Future work should aim to dissect the cell-type-specific roles of MOCCI in cell signaling and cell function, to better understand how mitochondria activity shapes glia biology to influence brain homeostasis and disease.

Given the field’s growing interest in mitochondrial modulation as a therapeutic strategy for neurodegeneration and inflammation, targeting MOCCI pharmacologically presents promising opportunities, allowing us to harness glial cells to promote neuroprotection and resolution in neuroinflammatory conditions. Because MOCCI is expressed in multiple cell types, systemic modulation could lead to unintended effects. Therefore, achieving precise tissue and cell type specificity would be key to maximize therapeutic benefits safely. Furthermore, chronic vs acute modulation, as well as varying degrees of modulations may lead to different outcomes. The complexity of the immune system means that precise and careful therapeutic targeting of MOCCI is needed to avoid compromising host defense, triggering unwanted inflammatory responses, and affecting mitochondrial function. Despite these challenges, the findings of this study lay the groundwork for understanding the role of MOCCI in neuroinflammation, providing a foundation for future studies to explore its mechanistic pathways and therapeutic potential in neurodegenerative and inflammatory brain disorders.

## Methods

### Mice

Pups at P1–4 were used to derive microglia and astrocytes, while adult mice between 10–14 weeks were used for the *in vivo* assays. To generate MOCCI-floxed allele, loxP sites were inserted before exon two and after exon four of MOCCI/AA467197 (ENSMUSG00000033213.16) gene. Founder mice were back-crossed for more than 8 generations. MOCCI-floxed homozygous mice were mated with Rosa-26-creERT2 mice to produce mice that excise MOCCI upon tamoxifen administration (**Figure 1a**). Pups were injected with 25 µL of 2 mg/mL 4-hydroxy-tamoxifen (Sigma Aldrich H6278) from P1-P3 and harvested on P4. Adult mice were fed via oral gavage with 50 µg tamoxifen/g mouse weight (Sigma Aldrich T5648) for 4 consecutive days. They were then rested for 1 week before they were used for experiments. To induce systemic inflammation, mice were injected intraperitoneally with 5 µg LPS/g mouse weight at 4 pm and harvested between 8-10am the next day.

Toe clips were obtained from mice younger than P10 and genotyped with the following primers: For cre (350bp): TGTTCAATTTACTGACCG and CGCCGCATAACCAGTGAAAC; To check for floxed (180bp) and WT (200bp) MOCCI allele: CATGTAGTCCTTGCACTTACAAAG and GCTCTAAGGGCAAGTTTTATTATCG.

### Immunoblot

Astrocytes and microglia were lysed in RIPA buffer with cOmplete™ EDTA-free Protease Inhibitor Cocktail (Roche 04693159001) and 1 mM phenylmethylsulfonyl fluoride (PMSF) (Sigma-Aldrich 10837091001). The lysates were pelleted at 20,000 rcf and the supernatant was quantified for protein concentration with BCA assay (ThermoFisher). Cell lysates were then normalized to equal concentrations, and boiled with 1X laemmli sample buffer (50 mM Tris-HCl pH 6.8, 2% SDS, 10% glycerol, 12.5 mM EDTA, 0.02% bromophenol blue, 50 mM DTT) at 95 °C for 10 min. Lysates were loaded into 4–12% gradient gels (ThermoFisher NW04122BOX), followed by transfer to a methonal-activated 0.2 µm PVDF membrane (ThermoFisher 22860). Blocking was done using 5% milk in TBST for 1 h at room temperature, with shaking. Membranes were probed with primary antibodies in in 5% milk in TBST overnight at 4 °C. Primary antibodies used were: anti-MOCCI (Novus Biologicals NBP1-98391; 1:500) and anti-GAPDH (Cell Signaling Technology 2118; 1:5000). Membranes were washed 3 times with TBST and probed with HRP/fluorescent protein conjugated secondary antibodies for 1 h at room temperature against rabbit IgG (Jackson ImmunoResearch 111-035-003; 1:4000) and washed 3 times with TBST. Signal was captured using the Chemidoc imaging system (Bio-Rad), and band intensities were quantified with Image Lab.

### Brain dissection and tissue dissociation

Mice were sacrificed by CO_2_ asphyxiation, and intracardial perfusion with ice-cold PBS was carried out to remove blood cells, until fluid runs clear (~20–30 mL). Mouse brains were dissected and either snap frozen in OCT in a dry ice and isopropanol bath for sectioning, or dissociated into single cells for scRNA-seq. Dissociation of brain tissue was done following manufacturer’s instructions with the Neural Dissociation Kit (Miltenyi Biotec). Briefly, the hippocampi from both sides were individually dissected and minced with a razor blade followed by enzymatic digestion for 20 min at 37 °C with shaking. The digested tissue was then gently triturated with a glass Pasteur pipette. The cell suspension was then strained through a 70 um cell strainer to remove tissue chunks, and the filtrate was centrifuged at 300g for 10 min to collect the cell pellet. The cell pellets were then re-suspended in Hibernate A (ThermoFisher).

### FACS sorting and cell hashing

Single-cell suspensions following tissue dissociation were resuspended in staining buffer (PBS + 2% FBS + 2 mM EDTA) and blocked with Fc receptor blocker (TruStain FcX™, BioLegend) for 30 minutes on ice, washed, and stained with unique TotalSeq™-A anti-mouse hashing antibodies (BioLegend 155831, 155833, 155835, 1558372 μg per 10^6^ cells) for 30 minutes at 4 °C, washed with 0.2% FCS/PBS, and spun down 300 g for 5 min at 4 °C. Secondary stained was done with anti-rat Alexa Fluor™ 647 (Thermo Fisher Scientific A48272, 1:1000) for 15 min on ice. After staining, cells were washed three times with 0.2% FCS/PBS and resuspended in Hibernate A. Equal numbers of live cells from each hashed sample were pooled, followed by staining with Propidium Iodide (PI) (Stemcell) before FACS sorting. Singlets, live cells (PI-negative) and Alexa Fluor 647–positive cells were sorted and processed for scRNA-seq.

### 10X library prep and scRNA-seq

For scRNA-seq, hippocampal tissues from both sides of one mouse per genotype/condition were used. Libraries from dissociated single cells were generated using Chromium Next GEM Single Cell 3’ Reagent Kits v3.2 (Dual Index) (10X Genomics). Cell numbers were counted by FACS and 8,000 cells per mouse were pooled after hashing, with a total of 32,000 cells used to generate libraries. Libraries were generated according to the manufacturer’s instructions (Chromium Single Cell 3’ Reagent Kits User Guide (v3.1 Chemistry) with Feature Barcoding technology for Cell Surface Protein). Feature barcoding was incorporated as BioLegend TotalSeq antibodies were used for cell hashing, where hashtag oligonucleotide (HTO) barcodes were added. Final libraries had a fragment length of 400–450 bp, and were sequenced on an Illumina NovaSeq 6000 using paired-end 150 bp (PE150) reads.

### scRNA-seq analysis pipeline

FASTQ files from 10X libraries were processed using Cell Ranger (10x Genomics) for alignment to the mouse reference genome (GRCm38). Filtered count matrices were analyzed using the Seurat package (version 5.3.0) in R (version 4.3.1). Briefly, QC was performed to keep only cells with percentage mitochondrial reads below 10%, and nFeature_RNA between 200 and 6000, to exclude empty droplets, multiplets and dying cells. Cells were normalized using SCTransform with mitochondrial gene percentage regressed out. Principal component analysis (PCA) was performed on the corrected data, and an elbow plot was used to determine the optimal number of dimensions. RunUMAP(), FFindNeighbors(), FindClusters() were run to generate the UMAP plots.

### Euclidean distance calculation

To assess transcriptional differences between annotated cell types and genotypes, Euclidean distances were computed based on average gene expression profiles. Cells were grouped by their assigned cell type, and the average expression for each gene within each group was calculated using the AverageExpression() function in Seurat (v5). Pairwise Euclidean distances were then computed between cell types using the dist() function. This distance matrix provided a quantitative measure of transcriptional dissimilarity across cell populations.

### Cluster proportion testing

Statistical differences in the proportion of microglial clusters were assessed using a permutation-based proportion test with the scProportionTest R package (v1.1.2). The sc_utils() function was first applied to the Seurat object to prepare the data, followed by permutation_test() to compare the proportions of clusters between WT-LPS and KO-LPS samples, using “seurat_clusters” as the cluster identity. The test evaluates whether any clusters are significantly over- or under-represented. Permutation_plot() was used to generate the summary plot for each cluster.

### DEGs and GSEA analysis pipeline

Differential expression (DE) analysis between KO-LPS and WT-LPS microglia was performed using the FindMarkers function in Seurat v5 with a Wilcoxon test. Only genes expressed in at least 20% of cells in either group were tested (min.pct = 0.2). p-values were adjusted using the Benjamini–Hochberg method to obtain false discovery rates (FDR). Genes with FDR < 0.05 and abs(log_2_FC) > 1 or 0.5 were considered significantly differentially expressed. Gene Ontology (GO) enrichment analysis was performed separately for genes upregulated in KO-LPS or WT-LPS conditions. GO enrichment was conducted using the enrichGO() function with the following parameters: ontology = “Biological Process”, pAdjustMethod = “BH”, and q-value cutoff of 0.05. Dot plots were generated to visualize the top 10 enriched terms.

### CellChat analysis

Cell-cell communication analysis was performed using the CellChat R package (v1.6.1), using the mouse ligand-receptor database (CellChatDB.mouse), with the standard analysis pipeline. First, a CellChat object was created using the single-cell dataset, grouped by cell type identity and genotype. The dataset was preprocessed with default parameters in the following order: identifyOverExpressedGenes(), identifyOverExpressedInteractions(), computeCommunProb(), filterCommunication(), computeCommunProbPathway(). The number and strength of interactions were visualized with circular plots and heatmaps. Role-specific signaling dynamics (incoming and outgoing interactions) were evaluated for each cell type and genotype. To examine condition-specific interactions, communication networks were subset to include only microglia and astrocyte populations, and the interaction networks were compared between C15orf48-WT and C15orf48-KO using compareInteractions().

### Primary astrocytes and microglia culture

Mouse brain cortices from P1–3 mice were dissected and placed into 2 mL of ice-cold DMEM per brain. Cortices were disrupted by gentle trituration using a 5 mL serological pipette 5 times, followed by gentle trituration using a glass Pasteur pipette 5 times. Disrupted tissue was then spun at 300 g for 5 min, and the pellet was resuspended in 1 mL of ice-cold DMEM. The cell suspension was filtered through a 40 mm cell strainer and cultured in 175 cm^2^ flasks in 10 mL DMEM with 10% FBS and 1% Pen-Strep. After 24h, the non-adherent cells and cellular debris were washed with 1X PBS and incubated with 20 mL of DMEM with 10% FBS and 1% Pen-Strep for 11–13 days. To obtain microglia, flasks were shaken for 1–2 h at 125 rpm in the incubator, and the culture media was collected and spun at 300 g for 5 min in FBS-coated 50 mL conical tubes. Microglia pellets were then resuspended, counted with Countess II (ThermoFisher) and plated. To obtain astrocytes, flasks were replenished with 10 mL DMEM with 10% FBS and 1% Pen-Strep and left to shake at 125 rpm overnight. Remaining adherent cells, which were mostly astrocytes, were trypsinized. Astrocytes were then spun down and resuspended to remove trypsin, counted with Countess II (ThermoFisher) and plated.

### Myelin isolation and labeling for phagocytosis assay

After CO_2_ asphyxiation, brains from adult mice were dissected and put through a discontinuous sucrose gradient to isolate myelin. Briefly, mouse brains were homogenized using a Dounce glass tissue grinder in ice-cold homogenization buffer (5mM HEPES(pH 7.4), 1mM MgCl2, 0.5mM CaCl2), and resuspended in 0.32M Tris-buffered sucrose. The homogenate was then layered over a sucrose gradient of 1.2M, 1.0M and 0.85M in an ultracentrifugation tube and ultracentrifuged at 82,500 g for 1 h at 4 °C in a swinging bucket rotor. Myelin collects at the interface between the 0.32M and 0.85M layers, and was carefully extracted using a needle and syringe. The myelin was then washed twice in distilled water (82,500 g for 15 min at 4 °C each wash) and resuspended in PBS, quantified with a BCA kit (ThermoFisher) and stored at −80 °C. Right before use, myelin was labeled with pHrodo Red Microscale Labeling Kit (Invitrogen) which allows it to be fluorescent only upon uptake into acidic environments (lysosomes).

### Phagocytosis assay

Phagocytosis assays with pHrodo Red-labeled myelin debris were performed using the Incucyte^®^ S3 instrument and software (Sartorius). 50,000 primary microglia or astrocytes were plated in each well of a 96-well plate the night before and allowed to adhere overnight. Cells were then pre-treated with IL-1β (10 ng/ml) or LPS (100 ng/ml) for 4–6 h prior to starting the assay. pHrodo Red-labeled myelin debris was added to each well (0 h timepoint) right before imaging. Red fluorescence and phase contrast images (10X objective) were acquired every hour for 8 hours, and a custom analysis pipeline within the Incucyte^®^ S3 software was used to quantify phagocytosed particles (red fluorescence).

### Scratch wound assay

Cell migration assays were performed with the Incucyte^®^ Scratch Wound Cell Migration Assay (Sartorius). 75,000 primary microglia and astrocytes were plated the night before on the Incucyte^®^ Image-Lock 96-well plate (cat 4379), and allowed to adhere. This ensures the cells reach full confluence the next day. Cells were then pre-treated with IL-1β (10 ng/ml) or LPS (100 ng/ml) 4–6 h prior to starting the assay. Next, a uniform scratch was created in all wells using the IncuCyte^®^ WoundMaker tool according to the manufacturer’s instructions (0 h timepoint). Detached cells were removed by washing twice with PBS, and fresh medium (with or without treatment) was added immediately. Phase contrast images (10X objective) were acquired every 2 h for 72 hours, and a custom analysis pipeline within the Incucyte^®^ S3 software was used to quantify relative wound density (RWD) over time. Specifically, the RWD is a metric that measures the density of the scratch wound region as a function of the density of the cell-occupied region.

### Luminex multiplexed cytokine assay

Primary microglia and astrocytes were seeded at 50,000 cells per well in 96 well plates and allowed to adhere overnight. Cells were then treated with IL-1β (10 ng/ml) or LPS (100 ng/ml) for 24 h in serum-free conditions. After 24 h, culture supernatants were collected and centrifuged at 1000g for 10 min to remove cellular debris. The supernatant was immediately stored at −80 °C, before Luminex assay. Luminex assay was performed following the manufacturer’s instructions using the Luminex Mouse 36 plex Exploratory Panel. Briefly, samples were thawed on ice. Standards and samples were then incubated with fluorescent magnetic beads conjugated to target-specific capture antibodies in a 96-well plate. The plates were read using a Luminex MAGPIX^®^ instrument (Luminex Corporation) and cytokine concentrations were calculated from standard curves with the Luminex software.

### Cuprizone chow model

8–16 week old mice were fed an irradiated diet containing 0.2% (w/w) cuprizone (0.2% bis(cyclohexanone)oxaldihydrazone) mixed into standard diet base (Envigo, TD.140803) for 4 weeks to induce demyelination in the corpus callosum. Diet was kept in a dark and airtight container at 4 °C and discarded 1 week after opening. After 4 weeks, mice were switched to normal chow to induce remyelination. Body weight and health were monitored twice weekly.

### MRI

Mice were placed in a dedicated induction chamber on a warm electric heating pad and anesthetized with 2-5% isoflurane in 1 L/min of O_2_ and medical grade air (ratio 1:1). They were then removed from the chamber, weighed and placed in custom designed cradle. Eye gel (Vidisic^®^ 0.2% w/w Eye Gel Carbome) was applied to both eyes to prevent drying. The temperature of animals was measured with a lubricated rectal temperature probe. Imaging was done using a 9.4 Tesla Bruker Biospec small-animal MRI system. During the imaging, animals received 1.5-4% isoflurane in in 1 L/min of O_2_ and medical grade air (ratio 1:1) via a nose cone. A respiratory monitor was used to monitor respiration throughout the procedure and levels of anesthetic were adjusted based on these readings. After imaging, the nose cone was removed and animals were allowed to recover on a warm electric heating pad. 0.2 mL of warm saline was given to each mouse subcutaneously to prevent dehydration. T2-weighted images covering the whole brain was generated with a RARE (Rapid Acquisition with Relaxation Enhancement) sequence with echo time (TE) = 33 ms. Data were analyzed in the lesion of the corpus callosum with ITK-SNAP v3.

### Immunofluorescence staining and imaging

OCT blocks stored at -80 °C were equilibrated at -20 °C for 2 h prior to sectioning, and cryosectioned at a thickness of 10 µm using a CM1950 cryostat. Sections were fixed with 4% vol/vol paraformaldehyde (PFA) for 1 h at room temperature, and washed four times in PBS to remove PFA. They were then permeabilized and blocked in 0.1% Triton X and 10% NGS in PBS. Sections were then incubated in primary antibodies diluted in 0.1% Triton X and 10% NGS in PBS overnight at 4 °C. Slides were then washed 3 x in PBS, and incubated with secondary antibodies diluted in 0.1% Triton X and 10% NGS in PBS for 2 h at room temperature. Slides were then washed 3 x in PBS, and incubated with DAPI stain (1:1000, ThermoFisher) for 5 min, followed by washing and mounting. The following primary antibodies were used: anti-Iba1 at 1:100 (Wako, 019-19741), anti-GFAP 1:50 (BioLegend, 5753). Alexa Fluor secondary antibodies were used at 1:500. Images were obtained with ZEISS Digital Slide Scanner Axioscan 7 using the Zen software.

### Particle morphometry with ImageJ analysis

Images were imported into ImageJ. First, scale of the image is set with the known actual distance. Next, images were converted to 8-bit and thresholded. Measurements were configured to Area, Shape descriptors, Integrated density, skewness, Area fraction, Fit ellipse, kurtosis and particles were quantified with “Analyze Particles”. Density of cells was calculated as the number of particles per mm^2^.

### Statistical analysis

Statistical analyses were performed using Prism 9 (GraphPad Software, San Diego, CA), R (version 4.3.1). Data are presented as mean ± standard error of the mean (SEM) unless otherwise indicated. Statistical significance was set at p = 0.05. For comparisons between WT and KO data were performed using unpaired two-tailed Student’s t-tests. For experiments with multiple treatment conditions (baseline, LPS, IL-1β), two-way ANOVA with genotype (WT vs KO), followed by Sidak’s multiple comparisons test for pairwise comparisons. For quantification of demyelinated lesion volume in MRI images was performed blinded to genotype using ITK-SNAP v3. Unpaired two-tailed Student’s t-tests was used to compare WT and KO measurements. Cell morphology and density were quantified using ImageJ particle analysis and morphometry.

## Data Availability

The processed single-cell gene expression data generated for this study have been deposited at the Gene Expression Omnibus (GEO) with the accession number GSE305874 and are publicly available. Gene expression data of the mouse cuprizone model used in this study were downloaded from GSE148676 and are publicly available. All R analysis codes used in this study are publicly available as part of the respective R packages cited. Additional information is available upon request.
