# Supplementary material for: Mitochondrial microprotein MOCCI controls neuroinflammation by altering glial activation states
Source: Front Immunol. 2026 Jul 9;17:1798798. doi: 10.3389/fimmu.2026.1798798 (PMC13391258; doi:10.3389/fimmu.2026.1798798)
Supplement: Supplementary file 1 [file DataSheet1.pdf]

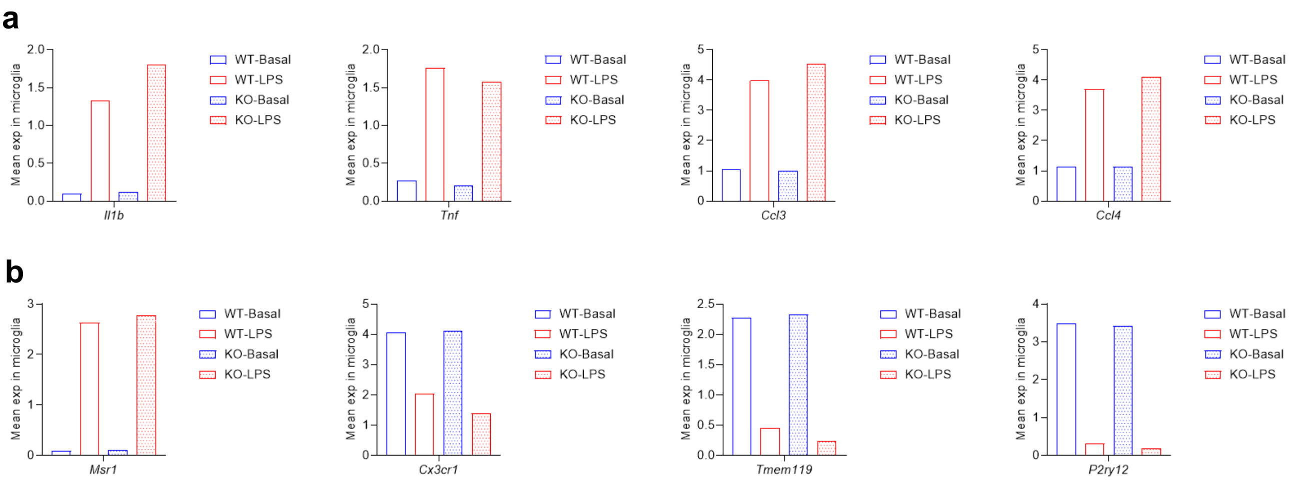

Fig. S1: MOCCI-KO microglia exhibit enhanced M1 pro-inflammatory markers and reduced homeostatic markers upon LPS stimulation.

(a) M1 pro-inflammatory markers (*Il1b, Ccl3, Ccl4*) are elevated in KO-LPS microglia compared to WT-LPS. *Tnf* expression, however, is decreased in KO-LPS compared to WT-LPS. (b) Homeostatic microglial markers (*Cx3cr1, Tmem119, P2ry12*) are reduced in KO-LPS microglia, while the M2 marker *Msr1* (CD206) shows elevated expression in LPS conditions, with slightly higher expression in KO-LPS. Data is mean expression from scRNA-seq transcriptomics data (n=1 per group).

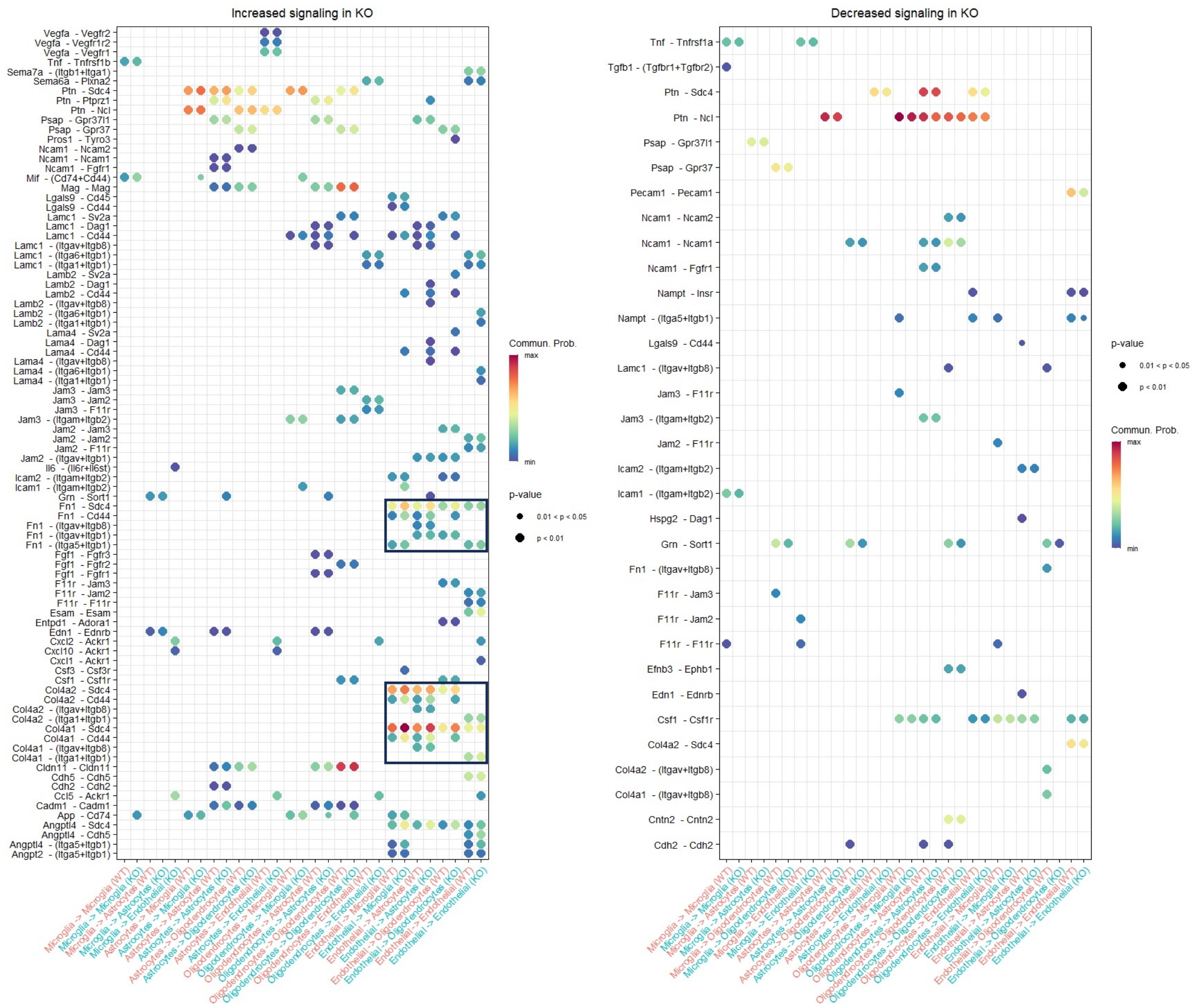

Fig. S2: CellChat analysis of up-regulated and down-regulated ligand-receptor pairs.

Comparison of communication probabilities by individual ligand–receptor pairs, showing cell-cell signaling changes caused by loss of C15orf48. Left: Ligand–receptor interactions with increased communication probability in KO compared to WT. Right: Ligand–receptor interactions with increased communication probability in WT compared to KO.

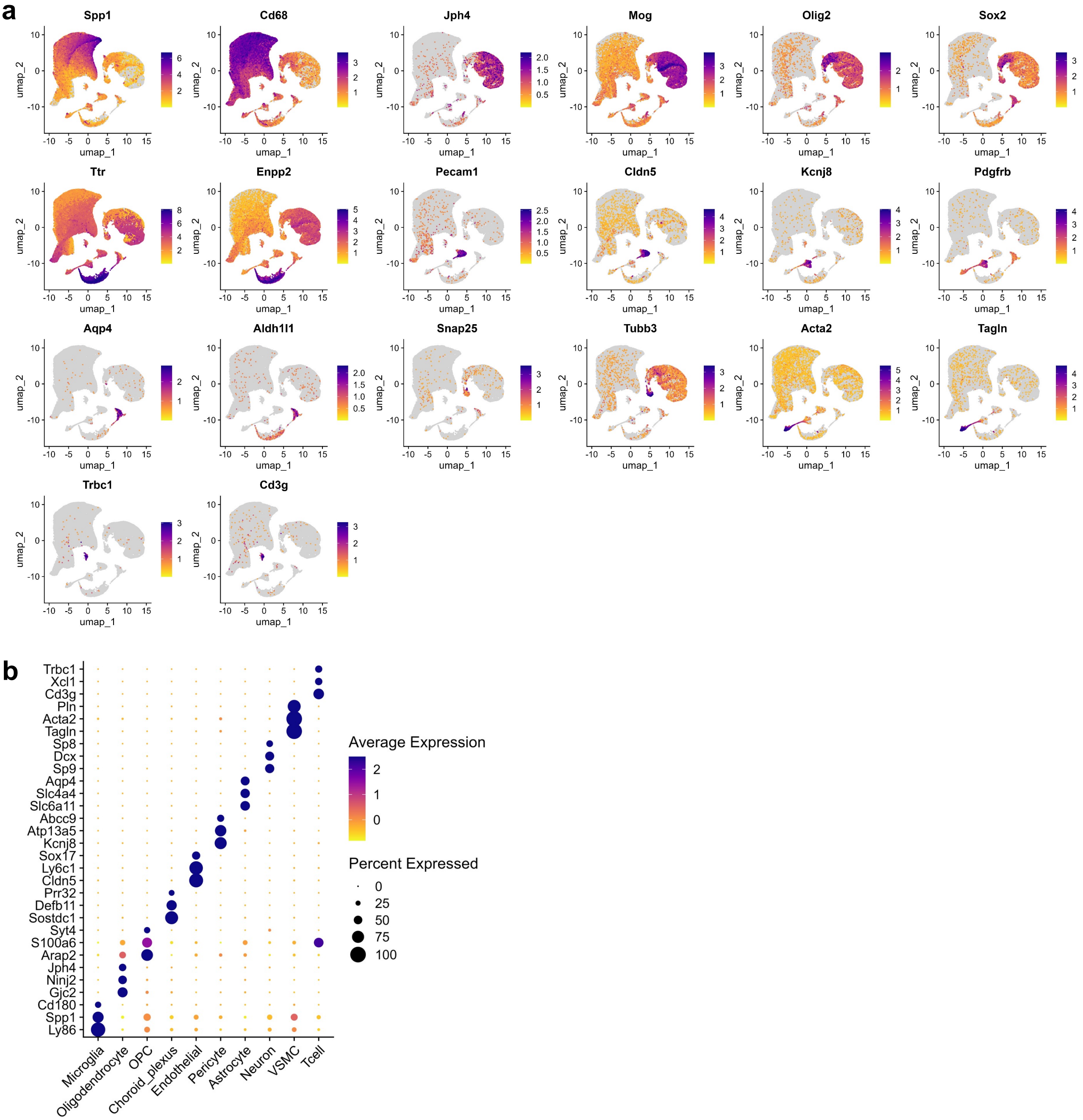

Fig. S3: Cell type annotation of a publicly available scRNA-seq dataset from a cuprizone mouse model of demyelination and remyelination.

(a) UMAP projection coloured by selected marker genes for cell type identification. Marker genes used are *Spp1, Cd68*: microglia; *Jph4, Mog*: oligodendrocytes; *Olig2, Sox2*: OPCs; *Ttr, Enpp2*: choroid plexus; *Pecam1, Cldn5*: endothelial cells; *Kcnj8, Pdgfrb*: pericytes; *Aqp4, Aldh1l1*: astrocytes; *Snap25, Tubb3*: neurons; *Acta2, Tagln*: VSMCs; *Trbc1, Cd3g*: T cells. (b) Dot plot showing top 3 marker genes in each annotated cell type.

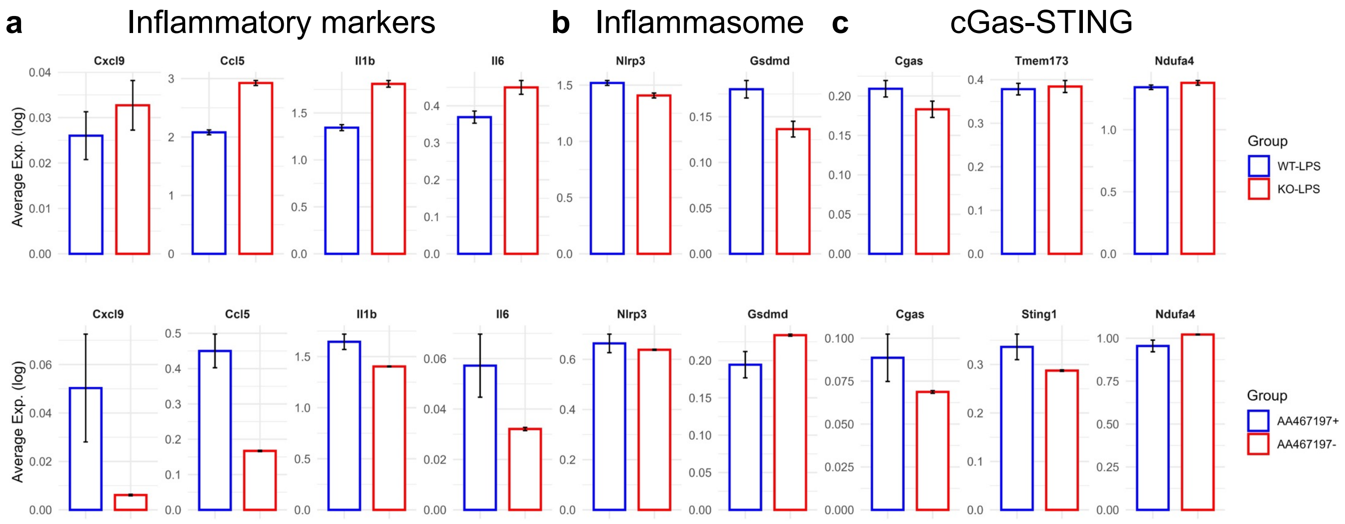

Fig. S4: Expression of inflammatory markers, inflammasome components, and cGAS–STING pathway genes in microglia.

Bar plots showing log-normalized counts of (a) inflammatory markers, (b) inflammasome components and (c) cGAS-STING pathway genes in in KO vs WT microglia from LPS-treated brains, and in AA467197-expressing vs non–AA467197-expressing microglia from cuprizone (4-week) brains. Error bars show standard error of the mean.
